# Application of gallbladder-cholangial plastic stent with a retraction thread in the treatment of acute calculous cholecystitis: the first clinical practice

**DOI:** 10.1055/a-2727-0139

**Published:** 2025-11-06

**Authors:** Rongjuan Zhu, Jiyu Zhang, Qingfen Zheng, Jie Liu, Bin Hai, Dan Liu, Bingrong Liu

**Affiliations:** 1 191599 Department of Gastroenterology and Hepatology, The First Affiliated Hospital of Zhengzhou University, Zhengzhou, China

A 55-year-old female was admitted to our hospital with intermittent abdominal pain for 8 months and aggravated for 1 day. Physical examination revealed a positive Murphy sign. CT scan indicated acute calculus cholecystitis ( Fig. 1 **a** ). The patient strongly desired gallbladder preservation. Considering the patient’s severe cholecystitis and increased risks of cholecystolithotomy, we concluded that controlling inflammation and relieving biliary obstruction should be the initial priorities. Therefore, we decided to perform cystic duct stent placement. ERCP showed no dilation of the common bile duct and non-visualization of the gallbladder ( Fig. 1 **b** ). Successful gallbladder cannulation and cholangiography were performed with a cholangioscope, which revealed multiple gallstones ( Fig. 1 **c, d** ). To protect gallbladder physiological function (maintaining hepatobiliary circulation, gallbladder storage, and bile excretion) and ensure controlled stent retrieval, we placed an improved new type of gallbladder-cholangial plastic stent (8.5-Fr 8 cm) with a retraction thread. Firstly, the side wings of the stent were removed, and a thread about 8 cm long was threaded at the distal end of the stent ( Fig. 2 ). The stent was then deployed into the biliary tract ( Video 1 , Fig. 1 **e, f** ). Postoperatively, the patient’s abdominal pain was relieved immediately and discharged. After 3 months, a CT scan revealed complete resolution of gallbladder wall edema, and the stent remained in situ ( Fig. 1 **g** ). Subsequent natural orifice transluminal endoscopic surgery, gallbladder-preserving cholecystolithotomy, and stent removal were performed successfully 1 . At 18-month follow-up, the patient was well ( Fig. 1 **h** ).

**Fig. 1:**
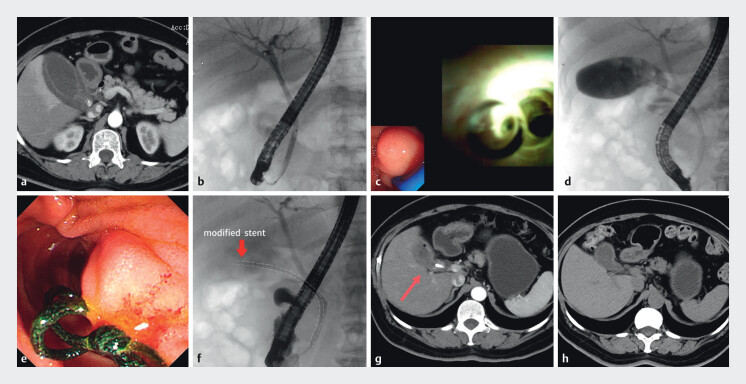
**a** The CT scan indicated a thickened and markedly edematous gallbladder wall. **b** ERCP showed no dilation of the common bile duct and non-visualization of the gallbladder. **c** Under direct visualization with the cholangioscope, the cystic duct was identified. **d** The cholecystography revealed multiple gallstones. **e** The end of the thread remained within the duodenal lumen. **f** The stent was then deployed into the biliary tract. **g** After 3 months, the CT scan showed complete resolution of gallbladder wall edema, and the stent remained in situ. **h** At 1-month follow-up, the CT scan showed complete clearance of gallstones without cholecystitis.

**Fig. 2:**
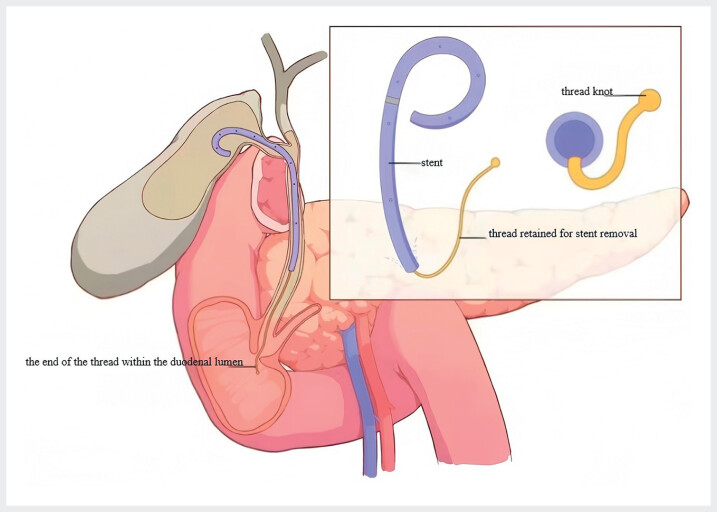
The proximal end of the stent was placed into the gallbladder cavity, with the distal end in the common bile duct, while the end of the thread remained within the duodenal lumen to ensure the stent could be removed.

Application of gallbladder-cholangial plastic stent with a retraction thread in the treatment of acute calculous cholecystitis.Video 1

For patients with acute calculous cholecystitis who request gallbladder preservation, cystic duct stent placement is effective to relieve biliary obstruction and alleviate gallbladder inflammation 2 3]. The application of a gallbladder-cholangial plastic stent with a retraction thread serves as a bridging therapy. It rapidly relieves biliary obstruction, protects gallbladder physiological function, and ensures easy stent removal. This approach paves the way for definitive surgery, whether gallbladder preservation or cholecystectomy.

Endoscopy_UCTN_Code_TTT_1AR_2AH

## Notes

Endoscopy E-Videos https://eref.thieme.de/e-videos
*E-Videos* is an open access online section of the journal *Endoscopy* , reporting on interesting cases and new techniques in gastroenterological endoscopy. All papers include a high-quality video and are published with a Creative Commons CC-BY license. Endoscopy E-Videos qualify for HINARI discounts and waivers and eligibility is automatically checked during the submission process. We grant 100% waivers to articles whose corresponding authors are based in Group A countries and 50% waivers to those who are based in Group B countries as classified by Research4Life (see: https://www.research4life.org/access/eligibility/ ). This section has its own submission website at https://mc.manuscriptcentral.com/e-videos .

## References

[1] Ullah S Yang BH Liu D Are laparoscopic cholecystectomy and natural orifice transluminal endoscopic surgery gallbladder preserving cholecystolithotomy truly comparable? A propensity matched study World J Gastrointest Surg 2022 14 470 481 10.4240/wjgs.v14.i5.470 35734621 PMC9160690

[2] Higa JT Irani SS Endoscopic Methods for Gallbladder Drainage Curr Treat Options Gastroenterol 2019 17 357 366 10.1007/s11938-019-00243-4 31317444

[3] Higa JT Sahar N Kozarek RA EUS-guided gallbladder drainage with a lumen-apposing metal stent versus endoscopic transpapillary gallbladder drainage for the treatment of acute cholecystitis (with videos) Gastrointest Endosc 2019 90 483 492 31054909 10.1016/j.gie.2019.04.238

